# An evaluation of statistical methods for DNA methylation microarray data analysis

**DOI:** 10.1186/s12859-015-0641-x

**Published:** 2015-07-10

**Authors:** Dongmei Li, Zidian Xie, Marc Le Pape, Timothy Dye

**Affiliations:** 1 Clinical and Translational Science Institute, School of Medicine and Dentistry, University of Rochester, 265 Crittenden Boulevard CU 420708, Rochester, 14642 NY USA; 2 Department of Biostatistics and Computational Biology, University of Rochester, 265 Crittenden Boulevard CU 420708, Rochester, 14642 NY USA; 3 John A. Burns School of Medicine, University of Hawaii, 651 Ilalo Street 101, Honolulu, 96813 HI USA; 4 Department of Obstetrics and Gynecology, University of Rochester, 500 Red Creek Drive Suite 220, Rochester, 14623 NY USA

**Keywords:** DNA methylation, Power, Stability

## Abstract

**Background:**

DNA methylation offers an excellent example for elucidating how epigenetic information affects gene expression. *β* values and *M* values are commonly used to quantify DNA methylation. Statistical methods applicable to DNA methylation data analysis span a number of approaches such as Wilcoxon rank sum test, *t*-test, Kolmogorov–Smirnov test, permutation test, empirical Bayes method, and bump hunting method. Nonetheless, selection of an optimal statistical method can be challenging when different methods generate inconsistent results from the same data set.

**Results:**

We compared six statistical approaches relevant to DNA methylation microarray analysis in terms of false discovery rate control, statistical power, and stability through simulation studies and real data examples. Observable differences were noticed between *β* values and *M* values only when methylation levels were correlated across CpG loci. For small sample size (*n*=3 or 6 in each group), both the empirical Bayes and bump hunting methods showed appropriate FDR control and the highest power when methylation levels across CpG loci were independent. Only the bump hunting method showed appropriate FDR control and the highest power when methylation levels across CpG sites were correlated. For medium (*n*=12 in each group) and large sample sizes (*n*=24 in each group), all methods compared had similar power, except for the permutation test whenever the proportion of differentially methylated loci was low. For all sample sizes, the bump hunting method had the lowest stability in terms of standard deviation of total discoveries whenever the proportion of differentially methylated loci was large. The apparent test power comparisons based on raw *p*-values from DNA methylation studies on ovarian cancer and rheumatoid arthritis provided results as consistent as those obtained in the simulation studies. Overall, these results provide guidance for optimal statistical methods selection under different scenarios.

**Conclusions:**

For DNA methylation studies with small sample size, the bump hunting method and the empirical Bayes method are recommended when DNA methylation levels across CpG loci are independent, while only the bump hunting method is recommended when DNA methylation levels are correlated across CpG loci. All methods are acceptable for medium or large sample sizes.

## Background

DNA methylation is a biochemical process of adding a methyl group at the $\phantom {\dot {i}\!}5^{'}$ carbon of the cytosine ring to form 5-methylcytosine (found at cytosine-guanosine dinucleotides (CpGs)) and plays a significant role in the development and progression of human disease [1]. More than 50 % of human gene transcription initiations are from genome regions with elevated CpG contents, known as “CpG islands”. CpG loci within promoter CpG islands are normally free from DNA methylation to allow the initiation of gene expression [2]. Studies have documented associations between DNA methylation and cancer [1, 3]. Promoter hypermethylation impacts development of cancer through transcriptional silencing of crucial growth regulators. Two United States Food and Drug Administration (FDA) approved epigenetic drugs, azacitidine and decitabine, reactivate tumor suppressor genes through removing DNA methylation marks, which highlights the importance of understanding DNA methylation in disease etiology and treatment [4, 5].

Large-scale examination of DNA methylation through microarray or sequencing technologies makes epigenome-wide association studies (EWAS) feasible to explore associations between DNA methylation and cancers in the sustained effort to develop novel anti-cancer drugs, and to identify DNA methylation markers associated with certain cancers for prognosis and diagnosis purpose [6]. The Illumina HumanMethylation BeadChip technology is a popular platform for conducting epigenome-wide association studies. Three platforms have been developed by Illumina for DNA methylation assay: GoldenGate, Infinium Human Methylation27 and Infinium HumanMethylation450 BeadChip. All platforms use two fluorescent dye colors to recognize the bisulphite-converted sequence. The standard output from the BeadChip assay for quantifying methylation is the *β* value, which is calculated from the intensity of methylated allele (*M* *a* *x*(*M*,0)) and the intensity of unmethylated allele (*M* *a* *x*(*U*,0)) according to the following formula [7].

(1) $$ \beta=\frac{Max(M, 0)}{Max(M, 0)+ Max(U, 0)+100}  $$

The *β* values are usually preprocessed for the downstream statistical analysis. The summary on preprocessing the *β* values including quality control, background correction, and normalization could be found somewhere else [8]. For differential DNA methylation analysis, the average *β* value denotes the methylation level, or the percentage for an interrogated locus. The average *β* values vary between 0 and 1. In an ideal situation, "zero" indicates that no copy of the CpG site in the sample is methylated, and "one" indicates that every copy of the site is methylated. The average *β* value approximates the methylation percent for the population of a sampled CpG site. Alternatively, some investigators use the *M*-value, considering the *M*-value alternative statistically more valid [9]. The *M*-value is defined as:

(2) $$ M={log}_{2}\frac{Max(M, 0)+1}{Max(U, 0)+1}.  $$

The range of *M*-values could be from −*i* *n* *f* to +*i* *n* *f*, consistent with the data range for a normal distribution. However, interpretations of *M*-values are not as intuitive as for *β*-values. A properly normalized *M*-value approaching zero indicates that a specific CpG site is half-methylated. Positive *M*-values suggest a methylation rate greater than 50 %, while negative *M*-values indicate a methylation inferior to 50 %. The *β*-values and *M*-values are related through a log2 ratio transformation such as:

(3) $$ M={log}_{2}\frac{\beta}{1-\beta}  $$

It has been shown that there is an approximately linear relationship between *β*-values and *M*-values in the middle range of the methylation data ([0.2, 0.8] for *β* values, and [-2, 2] for *M*-values) [9].

We used both *β*-values and *M*-values in our simulation studies and real data examples, which should provide guidance to investigators in selecting *β*-values or *M*-values for their differential DNA methylation analysis with regard to FDR control, power, and stability.

Currently available methylation differential analysis methods implemented in Bioconductor/R include several approaches such as Wilcoxon rank sum test (used in methyAnalysis package), *t*-test (used in methyAnalysis, CpGAssoc, RnBeads, and IMA package), Kolmogorov-Smirnov Tests (although not implemented in packages, but used by some investigators [10]), permutation test (used in CpGAssoc package), empirical Bayes method (used in RnBeads, IMA and minfi package), and bump hunting method (used in bumphunter and minfi package). However, with so many options available to investigators, selection of an optimal statistical method, can be challenging-especially when different methods applied to the same data set generate inconsistent results. As such, we systematically investigated these commonly used DNA methylation differential analysis methods in terms of their FDR control, power, and stability, through simulation studies. We illustrated the respective advantages and disadvantages of these methods with real methylation data sets, in order to provide empirical evidence and advice to investigators in selecting the most appropriate DNA methylation analysis methods for their studies.

## Methods

Hypothesis testing for each DNA methylation locus was done using either the average *β*-values or the transformed *M*-values of the different groups. Assume there are *m* methylation loci from the DNA methylation array assay. Among the *m* methylation loci, *m*_0_ loci are not differentially methylated. Suppose *R* methylation loci are rejected of *m* total loci, then *V* indicates the number of falsely rejected methylation loci (or “false discoveries”) from *R* rejections, and *S* denotes the true number of differentially methylated loci between groups in *R* rejections (*R*=*V*+*S*). The possible outcomes of testing *m* DNA methylation loci simultaneously are shown in Table 1. When testing *m* DNA methylation loci simultaneously, we need to control multiple testing error rate as opposed from testing a single DNA methylation locus.

**Table 1:** Possible outcomes from *m* hypotheses tests

	Number not rejected	Number rejected	
true null hypotheses	*U*	*V*	*m*_0_
non-true null hypotheses	*T*	*S*	*m*−*m*_0_
total	*m*−*R*	*R*	*m*

The most commonly used multiple testing error rate for discovery purposes is the false discovery rate proposed by Benjamini and Hochberg [11], defined as:

(4) $$ FDR = E\left(\frac{V}{R}|R > 0\right)Pr(R > 0).  $$

Another definition of false discovery rate proposes to control the expected proportion of false discoveries $E(\frac {V}{R}|R > 0)$ when *R*>0 [12] such as:

(5) $$ pFDR = E\left(\frac{V}{R}|R > 0\right).  $$

*p* *F* *D* *R*=1 when *m*_0_=*m*. FDR and pFDR set to 0 when *R*=0. FDR and pFDR are similar when the phenotype is not associated with DNA methylation for most of the CpGs.

In our simulations, two different multiple testing procedures were used to control for FDR/pFDR. Through a step-up procedure, the Benjamini-Hochberg procedure [11] provides control of FDR at *α* level. The Benjamini-Hochberg procedure compares ordered *P*_(*i*)_ with $\frac {i}{m}\alpha $ from the largest *p*, rejecting all *H*_(*i*)_ *i*=1,2,…,*k* with ${P_{(i)} \le \frac {i}{m}\alpha }$. The Benjamini-Hochberg procedure provides strong control for FDR at level *α* (for independent and positively correlated test statistics). Similarly to the Benjamini-Hochberg procedure, the Storey’s *q*-value procedure [12] uses conservative point estimators of *m*_0_, $\hat {m}_{0}(\lambda)$ (*λ* is a tuning parameter). With larger cutoffs, the Storey’s *q*-value leads to higher power than the Benjamini-Hochberg procedure as ${\hat {m}_{0}(\lambda) \leq m}$. The Storey’s *q*-value controls pFDR at *α*, with test statistics correlated weakly or independently.

Besides controlling FDR at a desired *α* level in the multiple testing process, we would also desire that the DNA methylation analysis method possess enough power to detect true differential DNA methylation loci and be consistent from experiment to experiment. Power is defined as the expected proportion of true differentially methylated loci detected among the total number of true differentially methylated loci [13]. Stability is measured as the standard deviation (SD) of the count of the differentially methylated loci detected. Power and stability of a differential DNA methylation analysis method could be expressed using the following formulas:

(6) $$\begin{array}{*{20}l} Power &= E\left(\frac{S}{m-m_{0}}|m > m_{0}\right), \end{array} $$

(7) $$\begin{array}{*{20}l} Stability &= SD(R). \end{array} $$

Power is defined as 0 and Stability becomes a measure of standard deviation of false detections when *m*=*m*_0_.

### Wilcoxon rank sum test (rank test)

Wilcoxon rank sum test (i.e., Mann–Whitney U test) is a rank-based non-parametric test and used in the methyAnalysis package as a differential methylation analysis method [14]. It is usually used as an alternative to the two-independent sample *t*-test when the assumption of normal data distribution is violated for the *t*-test.

Assume the methylation level denoted either by *β*-values or *M*-values for *i*th locus, *j*th group, and *k*th subject is *y*_*ijk*_. Suppose *j*=1 denotes the normal group and *j*=2 denotes the cancer group. For each DNA methylation locus, the null hypothesis of the Wilcoxon rank sum test is that the distribution of *y*_*i*1*k*_ equals the distribution of *y*_*i*2*k*_ for *i*=1,2,…,*m*. The two-side alternative hypothesis is a location shift of the distribution of *y*_*i*2*k*_ from *y*_*i*1*k*_ in either direction. The raw *p*-values from the Wilcoxon rank sum test are then adjusted using Benjamini and Hochberg procedure to control for FDR at level *α* [11] through the p.adjust function in R.

### ***t***-test

Implemented in methyAnalysis, CpGAssoc, RnBeads, and IMA packages, the *t*-test is a commonly used hypothesis testing method in genomic data analysis for testing equivalence of means between two groups [15]. For two independent samples *t*-test, there are two *t*-test procedures depending on whether the variances from those two groups are equal or not. The unequal variance *t*-test procedure (i.e., Welch’s *t*-test) is usually the default one used in most packages, and does not assume equal variance between groups. The raw *p*-values from the *t*-tests are computed based on the *t* distribution, while adjusted *p*-values are obtained using the Benjamini and Hochberg procedure through the same p.adjust function in R.

### Kolmogorov-Smirnov test (KS test)

The Kolmogorov-Smirnov test (KS test) is a nonparametric test in statistics for testing the equality of two continuous probability distributions [16, 17]. In DNA methylation studies, the null hypothesis is that the distribution of *y*_*i*1*k*_ equals the distribution of *y*_*i*2*k*_ as that in Wilcoxon rank sum test for each locus of *i*=1,2,…,*m*. Sensitive to difference in shape and location of the distribution functions of two groups, the KS test differs from the Wilcoxon rank sum test (sensitive to differences in location). The raw *p*-value from the KS test are adjusted using the Benjamini and Hochberg procedure to control FDR at level *α*.

### Permutation test

Permutation test is a resampling-based nonparametric test, which permutes data falling under the null hypothesis of equal data distributions between groups [18]. The distributions of test statistics (usually *t*-test statistics) are estimated from permuted test statistics. In the CpGAssoc package, the raw *p*-values from the permutation test for DNA methylation data are adjusted using the p.adjust function in R to control FDR at level *α*.

### Empirical Bayes method

Used in RnBeads, IMA and minfi packages, the empirical Bayes method is a popular hypothesis testing method applied through the lmFit and eBayes functions [19]. First, we can fit a linear model to estimate $\beta ^{*}_{i}$, the mean differences between two groups for *i*th locus. In DNA methylation studies, let ${y_{i}^{T}}=(y_{i_{1}}, \dots, y_{i_{n}})$ denote the DNA methylation level for both groups with *n*=*n*_1_+*n*_2_ for *i*th locus. Then, we can fit a linear model for each locus using the formula:

(8) $$ E(y_{i})=X\beta^{*}_{i},  $$

where *X* is a design matrix of full column rank, and $\beta ^{*}_{i}$ is a coefficient vector. The $\beta ^{*}_{i}$ coefficient vector includes $(\beta ^{*}_{i0}, \beta ^{*}_{i1})$ with $\beta ^{*}_{i0}$ denoting the mean DNA methylation level for normal group, while $\beta ^{*}_{i1}$ denotes the mean methylation level difference between the cancer group and the normal group. Thus, the null hypothesis for testing the mean methylation level difference between the normal group and the cancer group is $H_{0}: \beta ^{*}_{i1} = 0$ for locus *i*=1,…,*m*. The test statistic for testing *H*_0_ is the moderated *t*-statistic, based on a hybrid classical/Bayes approach, defined by:

(9) $$ \tilde{t}_{ij}=\frac{\hat{\beta}^{*}_{ij}}{\tilde{s}_{i} \sqrt{v_{ij}}}.  $$

The *p*-value for testing $H_{0}:\beta ^{*}_{\textit {ij}}=0$ ($H_{0}:\beta ^{*}_{i0}=0$ and $H_{0}:\beta ^{*}_{i1}=0$) based on the moderated *t*-statistic is calculated from the *t* distribution with *d*_*i*_+*d*_0_ degrees of freedom. More information on $\tilde {s}_{i}$, *v*_*ij*_, *d*_*i*_, and *d*_0_ could be found elsewhere [19].

The *p*-value for testing $H_{0}:\beta ^{*}_{i1}=0$ can be further adjusted using the p.adjust function to control for FDR at level *α*.

### Bump hunting method

The bump hunting method used in bumphunter and minfi packages was developed to take into account the correlations of methylation levels between nearby CpG locus [20]. The bump hunting method was carried out by first fitting a linear regression model for each locus before smoothing the coefficient within clusters along the genome to identify bumps [21]. More specifically, for each locus, a linear model will be used to estimate the coefficient of difference in methylation levels between the cancer group and the normal groups. Let *Y*_*ijk*_ denote the measured methylation level for *i*th locus, *j*th group, and *k*th subject. *X*_*ij*_ is an indicator variable with *X*_*i*1_=0 for the normal group and *X*_*i*1_=1 for the cancer group at all locus *i*. $\beta ^{*}_{i}$ is the estimated coefficient for *X*_*ij*_, and also stands for the estimated difference in DNA methylation levels between the cancer and the normal groups. We have then the following linear regression model.

(10) $$ Y_{ijk}=\mu_{i}+\beta^{*}_{i}X_{ij}+\epsilon_{ijk}  $$

where *ε*_*ijk*_ is the error term in the model, which follows a normal distribution with mean =0 and variance $={\sigma ^{2}_{i}}$.

After fitting the linear regression model, the bump hunting method will be implemented according to the following steps:

- Estimate $\beta ^{*}_{i}$ for each locus *i*.
- Estimate a smooth function *β* ^∗^(*t*) using these estimates.
- Use this smooth function *β* ^∗^(*t*) to estimate the regions *R*_*n*_, *n*=1,…,*N* for which *β* ^∗^(*t*)≠0 for all *t*∈*R*_*n*_.
- Assign statistical uncertainty to each estimated region using permutation tests.

We examined two *p*-values generated from the bump hunting method in the minfi package. One *p*-value is the raw *p*-value from the bump hunting method, adjusted through the Benjamini and Hochberg procedure using the p.adjust function in R (Bump hunting BH), and the other *p*-value is the *q*-value - an adjusted *p*-value generated from the minfi package using Storey’s procedure (Bump Hunting *q*-value) [22].

### Data extraction

We downloaded the ovarian cancer data set [23] and the rheumatoid arthritis data set [24] from the National Center for Biotechnology Information (NCBI) Gene Expression Omnibus (GEO) public functional genomics data repository. The ovarian cancer data set on 540 whole blood samples has GEO accession number GSE19711, generated from the Illumina Infinium 27k Human DNA methylation Beadchip v1.2. The rheumatoid arthritis data set on 691 subjects has GEO accession number GSE42861 and was generated using Illumina HumanMethylation450 BeadChip array. We randomly selected 3, 6, or 12 samples from either the case or the control groups to illustrate the apparent test power comparisons.

## Results

### Simulation study

We conducted simulation studies to compare the power and stability of six DNA methylation differential analysis methods for independent and correlated DNA methylation levels across CpG loci. Each simulation study included 1,000 independently generated two group samples with sample size (*n*) of 3, 6, 12, or 24 in both the cancer and normal groups. For all simulations, we set the total number of DNA methylation loci (*m*) as 1000. The fractions of truly differentially methylated loci $\left (\pi _{1} = \frac {m-m_{0}}{m}\right)$ were set at 1 %, 5 %, 10 %, 25 %, 50 %, 75 %, or 90 % to cover different scenarios. To mimic the data distribution of a real DNA methylation array experiment, the *β* values from the DNA methylation array studies for both cancer and normal groups were generated from a mixed beta distribution (0.1*B* *e* *t* *a*(0.5,5)+0.9*B* *e* *t* *a*(5,0.5)), for independent DNA methylation levels across CpG loci. For correlated DNA methylation levels across CpG loci, the *β* values *y*_*ij*_ for *i*th locus and *j*th subject were generated from $y_{\textit {ij}} = \frac {2^{log2(b_{\textit {ij}}) - log2(1-b_{\textit {ij}}) + e_{\textit {ij}}}}{1 + 2^{log2(b_{\textit {ij}}) - log2(1-b_{\textit {ij}}) + e_{\textit {ij}}}}$ where *b*_*ij*_ and *e*_*ij*_ were the (*i*,*j*)th elements of *m*×2*n* matrixes of *B* and *E* respectively. *B* was simulated from a *B* *e* *t* *a*(0.1,0.1) distribution and *E* was an matrix of error following a factor-analytic structure *E*=*L* *U* ^*T*^+*Φ* [25]. *L*=*Z*×*Δ* in which *Z* (a *m*×4 factor loadings matrix) denoted methylation profiles for constituent cell types and *Δ*=*d* *i* *a* *g*(0.55,0.35,0.07,0.03)^*T*^ (a 4×4 factor scores diagonal matrix) denoted cell proportions through its diagonal elements. *U* was a 2*n*×4 matrix of latent effects, and *Φ* was a *m*×2*n* random error matrix. Both *Z* and *U* were simulated from *N*(0,1) distribution. *Φ* were simulated from *N*(0,*σ*_*i*_) with ${\sigma ^{2}_{i}} = 0.5 - \sum (diag(\Delta ^{2}))$ (i.e. the standard deviation of each value was $\sqrt {0.5}$, but the errors were correlated across CpG loci). To set up the mean *β* value differences between groups, all *β* values of 1 %, 5 %, 10 %, 25 %, 50 %, 75 %, or 90 % of 1000 CpG loci in normal group subtracted a sequential vector from 0.1 to 0.4 with a length of 10, 50, 100, 250, 500, 750, and 900. For instance, with 1 % true difference between groups, the first 10 rows of *β* values from the normal group will equal the original *β* values in the normal group, generated either from the mixed beta distribution 0.1*B* *e* *t* *a*(0.5,5)+0.9*B* *e* *t* *a*(5,0.5) or from $y_{\textit {ij}} = \frac {2^{log2(b_{\textit {ij}}) - log2(1-b_{\textit {ij}}) + e_{\textit {ij}}}}{1 + 2^{log2(b_{\textit {ij}}) - log2(1-b_{\textit {ij}}) + e_{\textit {ij}}}}$, subtracted the sequential vector with a length of 10 from 0.1 to 0.4, i.e. (0.10,0.13,0.17,0.20,0.23,0.27,0.30,0.33,0.37,0.40). The *M*-values were generated using the *l* *o* *g* *i* *t*2 transformation of the *β*-values $\left (M = log2\left (\frac {\beta }{1 - \beta }\right)\right)$ and the FDR level was set at 5 %.

### Simulation results

#### Independent cases

**Fig. 1:**
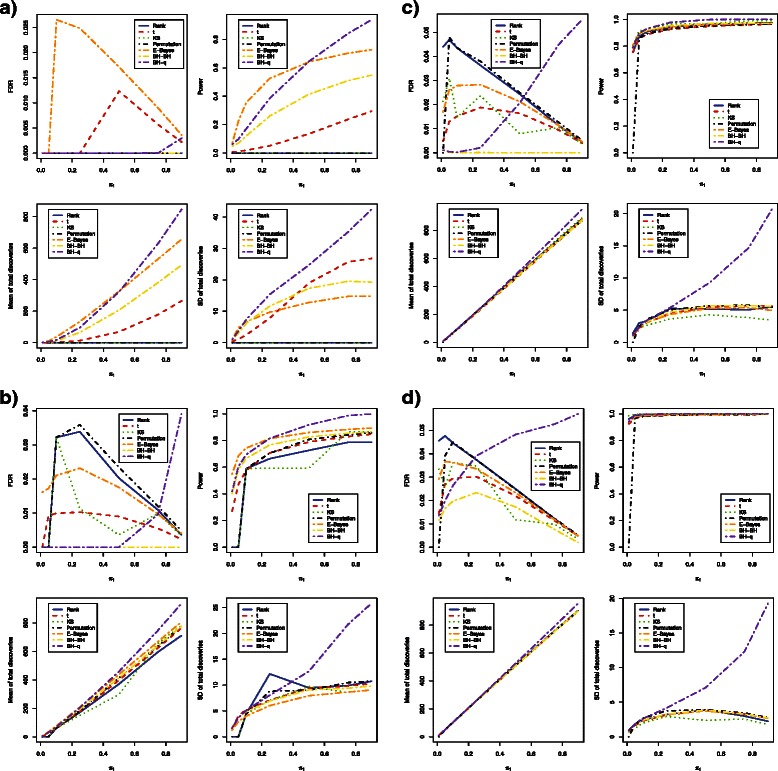
Estimated FDRs, powers, means of total discoveries, and SD of total discoveries of six DNA methylation differential analysis methods using *β* values (Independent case). Blue solid: rank test; Red dashed: *t*-test; Green dotted: KS test; Black dotdash: permutation test; Orange twodash: Empirical Bayes; Yellow twodash: Bump Hunting BH adjustment (BH-BH); Purple twodash: Bump Hunting *q*-value adjustment (BH-*q*)

**Fig. 2:**
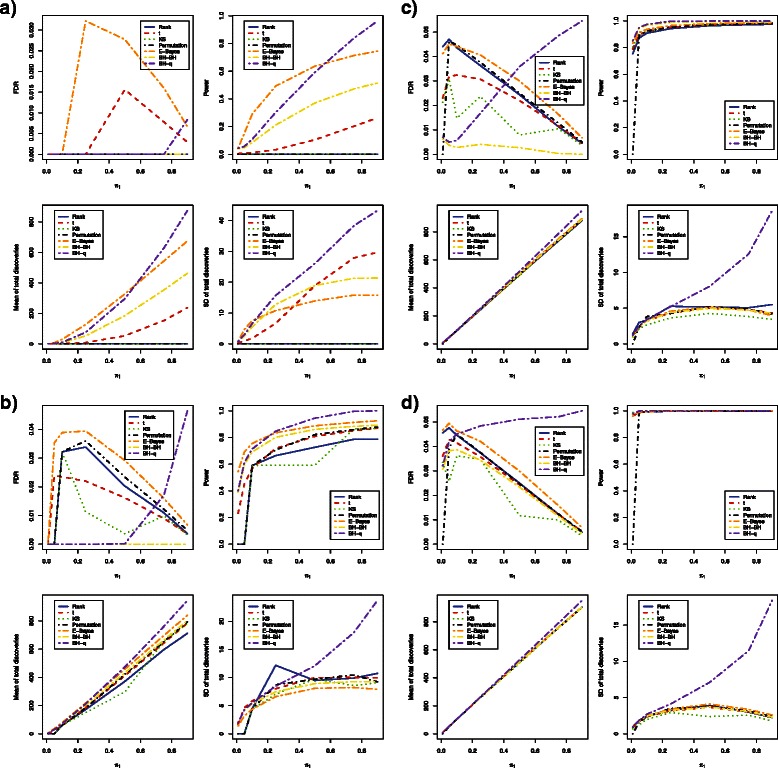
Estimated FDRs, powers, means of total discoveries, and SD of total discoveries of six DNA methylation differential analysis methods using *M* values (Independent case). Blue solid: rank test; Red dashed: *t*-test; Green dotted: KS test; Black dotdash: permutation test; Orange twodash: Empirical Bayes; Yellow twodash: Bump Hunting BH adjustment (BH-BH); Purple twodash: Bump Hunting *q*-value adjustment (BH-*q*)

For simulated DNA methylation data with sample sizes as small as 3 in each group, all methods could control FDR at a desired level of 5 % (Fig. 1 a and Fig. 2 a). In terms of power, the empirical Bayes method was the most powerful, followed by the bump hunting method and the *t*-test when the proportion of differentially methylated loci was below 50 % (Table 2 and Table 3). The bump hunting method was the most powerful method, followed by the empirical Bayes method and the t-test when the proportion of differentially methylated loci was above 50 %. Within the bump hunting method, power is higher with Storey’s *q*-value procedure than with Benjamini and Hochberg’s procedure. Neither the Wilcoxon rank sum test, the Kolmogorov-Smirnov test, nor the permutation test had power to identify any truly differentially methylated locus across all proportions of differentially methylated loci tested. For stability, the empirical Bayes method was much better than either the *t*-test or the bump hunting method (Table 4 and Table 5). The bump hunting method had the largest standard deviation of total discoveries once *p*-values were adjusted using Storey’s *q*-value procedure. The standard deviation of total discoveries from the bump hunting method increased exponentially as the proportions of differentially methylated loci increased. In the simulation studies, no significant differences were observed between *β* values and *M* values in terms of FDR control, power, mean number of total discoveries, or standard deviation of total discoveries.

**Table 2:** Powers across six DNA methylation differential analysis methods using *β* values for independent case

*n*	*π*_1_	Power						
		Rank test	*t*-test	KS test	Permutation	Empirical Bayes	Bump hunting BH	Bump hunting *q*-value
3	0.01	0	0.0071	0	0	0.0714	0.0492	0.0612
	0.05	0	0.0126	0	0	0.2278	0.0715	0.0993
	0.10	0	0.0197	0	0	0.3577	0.1212	0.1722
	0.25	0	0.0516	0	0	0.5252	0.2612	0.3861
	0.50	0	0.1358	0	0	0.6417	0.4144	0.6450
	0.75	0	0.2371	0	0	0.7027	0.5096	0.8414
	0.90	0	0.2945	0	0	0.7274	0.5487	0.9378
6	0.01	0.0000	0.2713	0.0000	0.0000	0.5483	0.4025	0.4180
	0.05	0.0000	0.4708	0.0000	0.0000	0.6856	0.5847	0.6045
	0.10	0.5894	0.5773	0.5894	0.5894	0.7441	0.6660	0.6953
	0.25	0.6640	0.7069	0.5926	0.7053	0.8147	0.7671	0.8164
	0.50	0.7263	0.7890	0.5919	0.8078	0.8603	0.8274	0.9175
	0.75	0.7865	0.8297	0.8654	0.8422	0.8834	0.8544	0.9874
	0.90	0.7875	0.8475	0.8666	0.8589	0.8943	0.8648	0.9997
12	0.01	0.7541	0.7523	0.8053	0.0000	0.8085	0.7838	0.7883
	0.05	0.8722	0.8581	0.9179	0.8607	0.8926	0.8898	0.8975
	0.10	0.9082	0.8884	0.9164	0.8932	0.9149	0.9191	0.9304
	0.25	0.9423	0.9258	0.9672	0.9311	0.9433	0.9546	0.9773
	0.50	0.9643	0.9488	0.9674	0.9520	0.9607	0.9703	0.9990
	0.75	0.9714	0.9599	0.9893	0.9619	0.9689	0.9719	1.0000
	0.90	0.9753	0.9646	0.9895	0.9663	0.9725	0.9700	1.0000
24	0.01	0.9612	0.9252	0.9875	0.0000	0.9364	0.9403	0.9417
	0.05	0.9883	0.9714	0.9973	0.9673	0.9779	0.9902	0.9917
	0.10	0.9929	0.9809	0.9991	0.9810	0.9855	0.9970	0.9980
	0.25	0.9967	0.9899	0.9997	0.9900	0.9923	0.9999	1.0000
	0.50	0.9982	0.9940	0.9997	0.9941	0.9955	1.0000	1.0000
	0.75	0.9989	0.9957	0.9999	0.9958	0.9967	0.9999	1.0000
	0.90	0.9990	0.9965	0.9999	0.9965	0.9973	0.9994	1.0000

**Table 3:** Powers across six DNA methylation differential analysis methods using *M* values for independent case

*n*	*π*_1_	Power						
		Rank test	*t*-test	KS test	Permutation	Empirical Bayes	Bump hunting BH	Bump hunting *q*-value
3	0.01	0	0.0051	0	0	0.0438	0.0399	0.0434
	0.05	0	0.0086	0	0	0.1527	0.0488	0.0573
	0.10	0	0.0126	0	0	0.2905	0.0848	0.1079
	0.25	0	0.0331	0	0	0.4927	0.2117	0.3023
	0.50	0	0.1051	0	0	0.6364	0.3702	0.5922
	0.75	0	0.2030	0	0	0.7135	0.4725	0.8386
	0.90	0	0.2633	0	0	0.7445	0.5148	0.9629
6	0.01	0.0000	0.2307	0.0000	0.0000	0.5442	0.3903	0.4004
	0.05	0.0000	0.4511	0.0000	0.0000	0.6911	0.5977	0.6131
	0.10	0.5894	0.5699	0.5894	0.5894	0.7561	0.6913	0.7140
	0.25	0.6640	0.7182	0.5926	0.7072	0.8352	0.8014	0.8476
	0.50	0.7263	0.8083	0.5919	0.8215	0.8876	0.8609	0.9450
	0.75	0.7865	0.8508	0.8654	0.8596	0.9133	0.8838	0.9964
	0.90	0.7875	0.8689	0.8666	0.8778	0.9254	0.8920	0.9999
12	0.01	0.7541	0.7896	0.8053	0.0000	0.8317	0.8475	0.8520
	0.05	0.8722	0.8956	0.9179	0.8910	0.9151	0.9447	0.9499
	0.10	0.9082	0.9224	0.9164	0.9240	0.9379	0.9682	0.9751
	0.25	0.9423	0.9535	0.9672	0.9565	0.9649	0.9890	0.9973
	0.50	0.9643	0.9708	0.9674	0.9730	0.9791	0.9944	1.0000
	0.75	0.9714	0.9784	0.9893	0.9799	0.9854	0.9919	1.0000
	0.90	0.9753	0.9817	0.9895	0.9829	0.9881	0.9878	1.0000
24	0.01	0.9612	0.9600	0.9875	0.0000	0.9597	0.9754	0.9763
	0.05	0.9883	0.9892	0.9973	0.9861	0.9905	0.9989	0.9990
	0.10	0.9929	0.9937	0.9991	0.9935	0.9946	0.9998	0.9999
	0.25	0.9967	0.9972	0.9997	0.9972	0.9977	1.0000	1.0000
	0.50	0.9982	0.9986	0.9997	0.9986	0.9990	1.0000	1.0000
	0.75	0.9989	0.9992	0.9999	0.9992	0.9994	1.0000	1.0000
	0.90	0.9990	0.9993	0.9999	0.9993	0.9995	1.0000	1.0000

**Table 4:** Standard deviation of total discoveries across six DNA methylation differential analysis methods using *β* values for independent case

*n*	*π*_1_	Standard deviation of total discoveries						
		Rank test	*t*-test	KS test	Permutation	Empirical Bayes	Bump hunting BH	Bump hunting *q*-value
3	0.01	0	0.39	0	0	1.02	0.87	1.03
	0.05	0	1.14	0	0	4.79	3.09	3.80
	0.10	0	2.48	0	0	6.67	6.2	7.45
	0.25	0	7.87	0	0	9.71	11.65	15.38
	0.50	0	18.97	0	0	12.78	17.29	24.73
	0.75	0	25.68	0	0	14.73	19.56	35.22
	0.90	0	26.8	0	0	14.82	19.28	42.44
6	0.01	0.00	1.61	0.00	0.00	1.30	1.67	1.70
	0.05	0.00	4.05	0.00	0.00	2.95	3.68	3.69
	0.10	4.44	5.22	4.44	4.44	4.02	4.8	5.13
	0.25	12.17	7.04	7.04	8.83	6.02	6.93	8.03
	0.50	9.47	9.42	9.73	9.01	7.94	9.08	12.65
	0.75	9.90	9.82	8.55	10.48	8.65	9.39	21.72
	0.90	10.73	10.28	9.10	10.77	9.05	9.84	25.79
12	0.01	1.41	1.03	1.02	0.00	0.99	1.10	1.11
	0.05	3.01	2.21	2.14	2.46	2.22	2.33	2.39
	0.10	3.32	3.09	2.66	3.58	3.11	3.21	3.42
	0.25	5.26	4.48	3.65	5.08	4.35	4.70	5.42
	0.50	5.16	5.53	4.28	5.66	5.30	5.71	9.26
	0.75	5.07	5.70	3.83	5.76	5.37	5.70	14.69
	0.90	5.51	5.60	3.46	5.47	4.95	5.56	20.73
24	0.01	0.96	0.77	0.71	0.00	0.85	0.87	0.91
	0.05	1.87	1.64	1.23	1.79	1.76	1.76	1.82
	0.10	2.42	2.30	1.98	2.70	2.38	2.40	2.62
	0.25	3.21	3.17	2.92	3.75	3.22	3.26	4.15
	0.50	3.89	3.78	2.38	3.93	3.74	3.85	7.08
	0.75	2.99	3.29	2.57	3.45	3.25	3.31	12.30
	0.90	2.24	2.71	1.77	2.80	2.66	2.72	19.26

**Table 5:** Standard deviation of total discoveries across six DNA methylation differentially analysis methods using *M* values for independent case

*n*	*π*_1_	Standard deviation of total discoveries						
		Rank test	*t*-test	KS test	Permutation	Empirical Bayes	Bump hunting BH	Bump hunting *q*-value
3	0.01	0	0.33	0	0	0.80	0.82	0.85
	0.05	0	0.92	0	0	4.66	2.64	2.95
	0.10	0	1.84	0	0	7.57	5.55	6.60
	0.25	0	6.64	0	0	10.79	12.82	15.70
	0.50	0	18.85	0	0	13.92	18.93	26.01
	0.75	0	27.87	0	0	15.81	21.28	38.30
	0.90	0	29.67	0	0	15.80	21.35	43.19
6	0.01	0.00	1.70	0.00	0.00	1.41	1.94	1.95
	0.05	0.00	4.61	0.00	0.00	3.19	4.20	4.26
	0.10	4.44	5.83	4.44	4.44	4.36	5.34	5.51
	0.25	12.17	8.01	7.04	8.71	6.60	7.46	8.47
	0.50	9.47	9.98	9.73	9.70	8.08	8.92	12.08
	0.75	9.90	10.02	8.55	10.46	8.23	9.28	18.02
	0.90	10.73	9.97	9.10	9.23	7.90	9.27	23.71
12	0.01	1.41	1.18	1.02	0.00	1.13	1.22	1.23
	0.05	3.01	2.36	2.14	2.35	2.39	2.45	2.51
	0.10	3.32	3.24	2.66	3.83	3.24	3.25	3.42
	0.25	5.26	4.36	3.65	4.26	4.58	4.40	5.20
	0.50	5.16	5.01	4.28	5.23	5.12	4.99	8.04
	0.75	5.07	4.80	3.83	4.88	4.75	4.74	12.59
	0.90	5.51	4.33	3.46	4.22	3.96	4.27	18.66
24	0.01	0.96	0.87	0.71	0.00	0.96	0.91	0.92
	0.05	1.86	1.72	1.23	1.63	1.82	1.75	1.84
	0.10	2.42	2.36	1.99	2.35	2.46	2.43	2.71
	0.25	3.21	3.18	2.92	3.50	3.40	3.22	4.09
	0.50	3.89	3.67	2.38	3.79	4.11	3.71	7.08
	0.75	2.99	3.04	2.57	3.14	3.37	3.07	11.45
	0.90	2.25	2.30	1.77	2.36	2.59	2.30	18.40

Increasing sample size to 6 in each group, the Wilcoxon rank sum test, the Kolmogorov-Smirnov test, and the permutation test all showed greater than zero power (Fig. 1 b and Fig. 2 b). While all methods could control FDR at 5 %, the empirical Bayes method remained the most powerful among all methods, followed by the bump hunting method and the *t*-test, when the proportion of differentially methylated loci was below 25 % (Table 2 and Table 3). The bump hunting method was the most powerful method, followed by the empirical Bayes method and the t-test/permutation test, whenever the proportion of differentially methylated loci was above 25 %. The power of the Wilcoxon rank sum test, the Kolmogorov-Smirnov test, and the permutation test was lower than the *t*-test whenever the proportion of differentially methylated loci was below 25 %; however, the power of the permutation test was similar to the *t*-test, whenever the proportion of differentially methylated loci was above 25 %. The Wilcoxon rank sum test and the Kolmogorov-Smirnov test had relatively lower power compared to the other methods, even after the proportion of differentially methylated loci increased to 25 % or higher. In terms of stability, the bump hunting method had the largest standard deviation of total discoveries and an exponentially increasing trend, especially when the proportion of differentially methylated loci was larger than 50 % (Table 4 and Table 5). All other methods showed similar stability, while the empirical Bayes method had relatively the smallest standard deviation of total discoveries across all proportions of differentially methylated loci. Significant differences were not observed between *β* values and *M*-values in terms of power, mean number of total discoveries, and standard deviation of total discoveries, except that the FDR was controlled at a lower level whenever the *β* values were used for the empirical Bayes method.

For a moderate sample size of 12 in each group, power was not significantly different across methods whenever the proportion of differentially methylated loci was greater than 1 % (Fig. 1 c and Fig. 2 c). The mean number of total discoveries was also similar. standard deviation of total discoveries was maintained at a relatively low level for all methods across all proportions of differentially methylated loci, except for the bump hunting method, which showed a relatively large standard deviation of total discoveries and an exponentially increasing trend, whenever the proportion of differentially methylated loci was above 25 % (Table 4 and Table 5). All methods still controlled FDR within a 5 % level and had a more conservative control of FDR as the proportion of differentially methylated loci increased, with the exception of the bump hunting method which was less conservative in FDR control as the proportion of differentially methylated loci increased. Aside from the fact that the FDR was controlled at a lower level whenever the *β* values were used for the empirical Bayes method, no significant differences were observed between *β* values and *M*-values in terms of power, mean number of total discoveries, and standard deviation of total discoveries.

Similar simulation results were observed when sample size was increased to 24 in each group (Fig. 1 d and Fig. 2 d). The power of all methods became almost identical, and the large standard deviation of the bump hunting method became more obvious. Whenever *β* values were used for analysis using the empirical Bayes method, the FDR was controlled at a relatively lower level as compared to using *M* values. No significant power or stability differences were observed between the *β* values and *M* values.

Overall, the power and stability of all methods increased as sample size increased for both *β* values and *M* values (Table 2, 3, 4 and 5). It was observed that the permutation method retained lower power whenever the proportion of differentially methylated loci was as low as 1 %, regardless of sample size. The Wilcoxon rank sum test and the Kolmogorov-Smirnov test had exactly the same power and stability whenever either *β* values or *M* values were used.

#### Correlated cases

**Fig. 3:**
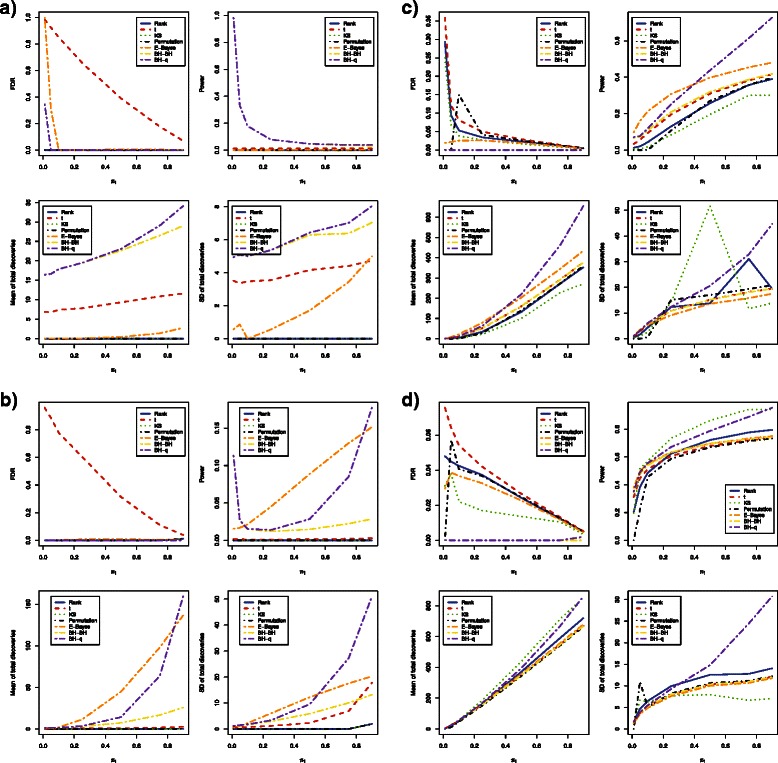
Estimated FDRs, powers, means of total discoveries, and SD of total discoveries of six DNA methylation differential analysis methods using *β* values (Correlated case). Blue solid: rank test; Red dashed: *t*-test; Green dotted: KS test; Black dotdash: permutation test; Orange twodash: Empirical Bayes; Yellow twodash: Bump Hunting BH adjustment (BH-BH); Purple twodash: Bump Hunting *q*-value adjustment (BH-*q*)

**Fig. 4:**
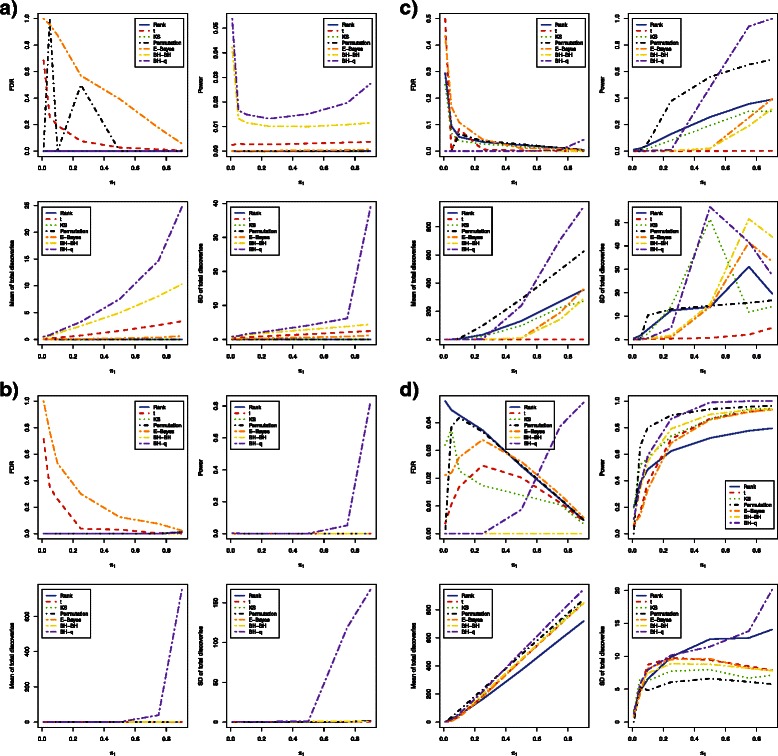
Estimated FDRs, powers, means of total discoveries, and variances of total discoveries of six DNA methylation differential analysis methods using *M* values (Correlated case). Blue solid: rank test; Red dashed: *t*-test; Green dotted: KS test; Black dotdash: permutation test; Orange twodash: Empirical Bayes; Yellow twodash: Bump Hunting BH adjustment (BH-BH); Purple twodash: Bump Hunting *q*-value adjustment (BH-*q*)

When methylation levels were correlated across CpG loci and sample size was as small as 3 in each group, the FDR and power estimates were different from independent cases. The *t*-test and empirical Bayes method had very large FDR estimates with both *β* values and *M* values. The bump hunting method had estimated FDR exceeding 0.05 when *β* values were used, but the FDR was well controlled at 5 % when *M* values were used (Fig. 3 a and Fig. 4 a). Interestingly, the bump hunting method had much higher power than all other methods especially when the proportion of differentially methylated loci was lower than 25 %. We also noticed that the power of the bump hunting method was higher when using *β* values than when using *M* values (Table 6 and Table 7). The bump hunting method also had a larger mean of total discoveries than all other methods, and identified more loci when *β* values were used. The stability trend remained the same as in the independent case. The bump hunting method still had the lowest stability among all methods compared (Table 8 and Table 9).

**Table 6:** Powers across six DNA methylation differential analysis methods using *β* values for correlated case

*n*	*π*_1_	Power						
		Rank test	*t*-test	KS test	Permutation	Empirical Bayes	Bump hunting BH	Bump hunting *q*-value
3	0.01	0	0.0112	0	0	0.0000	0.9854	0.9854
	0.05	0	0.0106	0	0	0.0002	0.3328	0.3328
	0.10	0	0.0113	0	0	0.0000	0.1788	0.1788
	0.25	0	0.0108	0	0	0.0003	0.0779	0.0779
	0.50	0	0.0113	0	0	0.0009	0.0453	0.0461
	0.75	0	0.0119	0	0	0.0018	0.0353	0.0388
	0.90	0	0.0120	0	0	0.0031	0.0322	0.0379
6	0.01	0.0000	0.0018	0.0000	0.0000	0.0154	0.1131	0.1135
	0.05	0.0000	0.0014	0.0000	0.0000	0.0167	0.0266	0.0270
	0.10	0.0000	0.0012	0.0000	0.0000	0.0215	0.0150	0.0153
	0.25	0.0000	0.0012	0.0000	0.0000	0.0450	0.0121	0.0139
	0.50	0.0000	0.0014	0.0000	0.0000	0.0889	0.0148	0.0285
	0.75	0.0000	0.0020	0.0000	0.0000	0.1300	0.0224	0.0851
	0.90	0.0001	0.0029	0.0001	0.0001	0.1514	0.0287	0.1773
12	0.01	0.0128	0.0333	0.0060	0.0000	0.0982	0.0676	0.0707
	0.05	0.0204	0.0564	0.0072	0.0000	0.1598	0.0726	0.0765
	0.10	0.0432	0.0934	0.0174	0.0002	0.2135	0.1085	0.1183
	0.25	0.1298	0.1953	0.0836	0.1070	0.3081	0.2091	0.2514
	0.50	0.2574	0.3095	0.1953	0.2701	0.3976	0.3195	0.4377
	0.75	0.3572	0.3824	0.3009	0.3582	0.4547	0.3874	0.6143
	0.90	0.3902	0.4170	0.3011	0.3975	0.4806	0.4186	0.7283
24	0.01	0.2030	0.3114	0.1950	0.0000	0.3806	0.3472	0.3515
	0.05	0.3841	0.4476	0.5185	0.2202	0.4968	0.4795	0.4890
	0.10	0.4865	0.5115	0.5711	0.4547	0.5510	0.5407	0.5560
	0.25	0.6240	0.6080	0.7351	0.5875	0.6333	0.6338	0.6718
	0.50	0.7222	0.6796	0.8634	0.6699	0.6968	0.6972	0.7876
	0.75	0.7769	0.7209	0.9425	0.7145	0.7335	0.7295	0.8899
	0.90	0.7954	0.7395	0.9431	0.7343	0.7503	0.7423	0.9548

**Table 7:** Powers across six DNA methylation differential analysis methods using *M* values for correlated case

*n*	*π*_1_	Power						
		Rank test	*t*-test	KS test	Permutation	Empirical Bayes	Bump hunting BH	Bump hunting *q*-value
3	0.01	0	0.0025	0	0	0.0000	0.0422	0.0539
	0.05	0	0.0030	0	0	0.0000	0.0133	0.0166
	0.10	0	0.0027	0	0	0.0001	0.0116	0.0149
	0.25	0	0.0027	0	0	0.0002	0.0101	0.0132
	0.50	0	0.0031	0	0	0.0003	0.0100	0.0151
	0.75	0	0.0035	0	0	0.0004	0.0108	0.0196
	0.90	0	0.0038	0	0	0.0007	0.0115	0.0275
6	0.01	0.0000	0.0002	0.0000	0.0000	0.0000	0.0040	0.0043
	0.05	0.0000	0.0003	0.0000	0.0000	0.0001	0.0013	0.0015
	0.10	0.0000	0.0002	0.0000	0.0000	0.0001	0.0008	0.0009
	0.25	0.0000	0.0002	0.0000	0.0000	0.0002	0.0007	0.0009
	0.50	0.0000	0.0002	0.0000	0.0000	0.0002	0.0006	0.0011
	0.75	0.0000	0.0003	0.0000	0.0000	0.0003	0.0007	0.0518
	0.90	0.0001	0.0003	0.0001	0.0001	0.0004	0.0007	0.8306
12	0.01	0.0125	0.0002	0.0060	0.0000	0.0013	0.0012	0.0015
	0.05	0.0201	0.0002	0.0071	0.0000	0.0008	0.0014	0.0017
	0.10	0.0429	0.0002	0.0172	0.0625	0.0008	0.0016	0.0021
	0.25	0.1291	0.0004	0.0828	0.3775	0.0013	0.0032	0.0099
	0.50	0.2566	0.0005	0.1935	0.5593	0.0194	0.0214	0.4811
	0.75	0.3560	0.0008	0.3004	0.6525	0.2526	0.1901	0.9410
	0.90	0.3894	0.0016	0.3010	0.6904	0.3945	0.3202	0.9993
24	0.01	0.2000	0.0602	0.1955	0.0000	0.0779	0.1065	0.1111
	0.05	0.3824	0.1561	0.5173	0.6587	0.1322	0.3315	0.3579
	0.10	0.4851	0.3883	0.5719	0.8010	0.3327	0.5460	0.5907
	0.25	0.6233	0.7138	0.7358	0.8928	0.6846	0.7914	0.8661
	0.50	0.7215	0.8666	0.8637	0.9394	0.8597	0.8997	0.9904
	0.75	0.7762	0.9197	0.9422	0.9577	0.9179	0.9326	1.0000
	0.90	0.7948	0.9380	0.9431	0.9649	0.9366	0.9407	1.0000

**Table 8:** Standard deviation of total discoveries across six DNA methylation differential analysis methods using *β* values for correlated case

*n*	*π*_1_	Standard deviation of total discoveries						
		Rank test	*t*-test	KS test	Permutation	Empirical Bayes	Bump hunting BH	Bump hunting *q*-value
3	0.01	0	3.51	0	0	0.57	4.94	4.94
	0.05	0	3.36	0	0	0.87	5.08	5.08
	0.10	0	3.48	0	0	0.00	5.01	5.01
	0.25	0	3.55	0	0	0.56	5.36	5.37
	0.50	0	4.17	0	0	1.71	6.30	6.43
	0.75	0	4.41	0	0	3.45	6.38	7.03
	0.90	0	4.74	0	0	5.00	7.04	8.05
6	0.01	0.00	0.70	0.00	0.00	0.41	1.26	1.26
	0.05	0.00	0.77	0.00	0.00	1.19	1.47	1.50
	0.10	0.00	0.72	0.00	0.00	2.26	1.68	1.73
	0.25	0.00	1.16	0.00	0.00	6.00	2.93	3.33
	0.50	0.00	2.32	0.00	0.00	12.26	5.89	9.53
	0.75	0.00	6.88	0.00	0.00	17.56	10.06	27.22
	0.90	1.94	17.78	1.94	1.97	20.27	13.17	51.04
12	0.01	0.51	0.94	0.29	0.00	1.07	1.06	1.11
	0.05	1.62	2.79	0.99	0.00	3.48	2.96	3.07
	0.10	4.32	5.61	2.48	0.63	5.38	5.74	6.06
	0.25	12.32	10.81	14.19	15.07	9.15	10.80	12.37
	0.50	13.79	15.16	51.68	17.03	13.59	15.14	20.58
	0.75	31.06	18.20	11.72	19.36	15.82	18.11	32.88
	0.90	19.40	19.47	13.90	20.84	17.52	19.47	44.66
24	0.01	1.66	1.62	1.67	0.00	1.35	1.62	1.62
	0.05	4.68	3.77	6.71	10.98	3.44	3.78	3.86
	0.10	6.50	5.31	6.31	5.09	4.91	5.32	5.47
	0.25	9.92	7.98	7.73	8.23	7.62	8.01	9.26
	0.50	12.56	10.37	7.95	10.64	10.00	10.40	14.80
	0.75	12.78	11.00	6.64	11.17	10.69	11.00	24.48
	0.90	14.06	12.13	7.07	12.19	11.78	12.13	30.75

**Table 9:** Standard deviation of total discoveries across six DNA methylation differentially analysis methods using *M* values for correlated case

*n*	*π*_1_	Standard deviation of total discoveries						
		Rank test	*t*-test	KS test	Permutation	Empirical Bayes	Bump hunting BH	Bump hunting *q*-value
3	0.01	0	0.30	0	0.00	0.22	0.76	0.88
	0.05	0	0.45	0	0.03	0.19	0.90	1.04
	0.10	0	0.67	0	0.00	0.26	1.35	1.61
	0.25	0	1.00	0	0.04	0.36	2.00	2.48
	0.50	0	1.56	0	0.03	0.62	2.93	4.13
	0.75	0	2.13	0	0.03	0.85	3.82	6.21
	0.90	0	2.48	0	0.03	1.18	4.46	38.95
6	0.01	0.00	0.08	0.00	0.00	0.14	0.21	0.21
	0.05	0.00	0.14	0.00	0.00	0.11	0.26	0.29
	0.10	0.00	0.17	0.00	0.00	0.14	0.29	0.31
	0.25	0.00	0.27	0.00	0.00	0.25	0.48	0.55
	0.50	0.00	0.39	0.00	0.00	0.36	0.67	0.96
	0.75	0.00	0.49	0.00	0.00	0.59	0.83	119.14
	0.90	1.94	0.54	1.94	1.97	0.91	0.98	166.82
12	0.01	0.50	0.06	0.29	0.00	0.16	0.12	0.13
	0.05	1.61	0.11	0.98	0.00	0.23	0.32	0.36
	0.10	4.29	0.16	2.47	10.35	0.35	0.52	0.60
	0.25	12.39	0.36	14.19	12.94	0.99	1.71	4.88
	0.50	13.82	0.68	51.43	14.47	13.96	15.04	56.63
	0.75	31.13	2.07	11.71	15.66	41.52	51.51	41.62
	0.90	19.59	4.98	14.03	16.74	33.38	43.90	27.44
24	0.01	1.65	0.83	1.67	0.00	0.89	1.24	1.28
	0.05	4.72	5.14	6.69	5.33	4.23	5.56	5.81
	0.10	6.54	8.72	6.29	4.82	8.17	7.44	7.95
	0.25	9.91	9.78	7.74	6.07	9.53	8.87	10.16
	0.50	12.61	9.43	7.97	6.60	9.63	8.78	11.47
	0.75	12.79	8.51	6.67	6.12	8.20	8.19	13.85
	0.90	14.08	7.87	7.14	5.77	7.84	7.81	20.16

When increasing sample size from 3 to 6 in each group, the FDR and power estimates also showed different characteristics from independent cases. The *t*-test still had very large FDR estimates when either *β* values or *M* values were used. The empirical Bayes method had decent control of FDR when *β* values were used; however, it lost control of FDR when *M* values were used (Fig. 3 b and Fig. 4 b). When *M* values were used, the bump hunting method had the highest power among all methods compared. When *β* values were used, the bump hunting method was the most powerful method, followed by the empirical Bayes method and the *t*-test whenever the proportion of differentially methylated loci was lower than 10 % or higher than 75 %, while the empirical Bayes method had the highest power whenever the proportion of differentially methylated loci was between 10 % and 75 % (Table 6 and Table 7). For stability, the bump hunting method had still the largest standard deviation of total discoveries whenever the proportion of differentially methylated loci was high, either with *β* values or *M* values (Table 8 and Table 9).

For a sample size of 12 in each group, the power and stability of all methods started to converge. The Wilcoxon rank sum test, the *t*-test, and the Kolmogorov-Smirnov test had estimated FDR values larger than 0.05 whenever *β* values were used and the proportion of differentially methylated loci was smaller than 10 %. When *M* values were used, only the bump hunting method and the permutation test had FDR controlled at 5 % whenever the proportion of differentially methylated loci was smaller than 10 % (Fig. 3 c and Fig. 4 c). With *β* values, the empirical Bayes method had slightly higher power than all other methods whenever the proportion of differentially methylated loci was smaller than 25 %. The bump hunting method had the highest power whenever the proportion of differentially methylated loci was greater than 25 %. Using *M* values, the permutation test had the highest power whenever the proportion of differentially methylated loci was smaller than 50 %, and the bump hunting method had the highest power whenever the proportion of differentially methylated loci was greater than 50 % (Table 6 and Table 7). The stability of all methods began to converge, but the bump hunting method still showed slightly larger standard deviation than all other methods compared (Table 8 and Table 9).

With a sample size of 24 in each group, the power of all methods was similar (Table 6 and Table 7). The *t*-test had estimated FDR over 5 % whenever *β* values were used and the proportion of differentially methylated loci was smaller than 10 %. All methods had estimated FDR within 5 % when *M* values were used (Fig. 3 d and Fig. 4 d). The bump hunting method with Storey’s *q*-value adjustment still showed low stability whenever the proportion of differentially methylated loci was large (Table 8 and Table 9).

In summary, the power and stability of all methods showed differences when using *β* values versus *M* values in all correlated cases (Table 6, 7, 8 and 9). Whenever sample sizes were 3, 6, or 12 in each group, the *t*-test, the empirical Bayes method, and the bump hunting method had larger power using *β* values than *M* values. The same observation was made whenever sample size was increased to 24 in each group with the proportion of differentially methylated loci smaller than 25 %. The Wilcoxon rank sum test and the Kolmogorov-Smirnov test had similar power using *β* values or *M* values, and the permutation test had higher power using *M* values than *β* values. The permutation method still retained low power whenever the proportion of differentially methylated loci was as low as 1 %, regardless of sample size. All methods were observed to produce slightly larger standard deviations whenever using *β* values rather than *M* values, except for the bump hunting method and the empirical Bayes method whenever the proportion of differentially methylated loci was larger than 50 % for sample size of 12 in each group.

### Real data examples

#### Ovarian cancer

Ovarian cancer ranks fifth in cancer death among women in the United States [26]. Aberrant DNA methylation was found to be associated with ovarian cancer. A genome wide DNA methylation profiling of United Kingdom Ovarian Cancer Population Study (UKOPS) was conducted to identify methylation signatures associated with carcinogenesis [23]. The data is available publicly, downloaded from the NCBI GEO website with GEO number GSE19711. The data originated from the Illumina Infinium 27k Human DNA methylation Beadchip v1.2 with 27578 CpGs from 540 whole blood samples, and 266 samples were taken from post-menopausal ovarian cancer patients, and 274 from normal controls (age-matched). To illustrate the differences in apparent test power (total number of discoveries) across the six methods at different FDR levels, we randomly selected either 3, 6, or 12 samples from both the cancer pre-treatment group and control group. The FDR levels ranged from 0.01 to 0.10, with a step of 0.01. Due to lack of significants using adjusted p-values for all methods, the raw *p*-values were used for comparisons. Thus, the Storey’s *q*-value procedure and the Benjamini-Hochberg procedure from the bump hunting method had the same raw *p*-values.

**Fig. 5:**
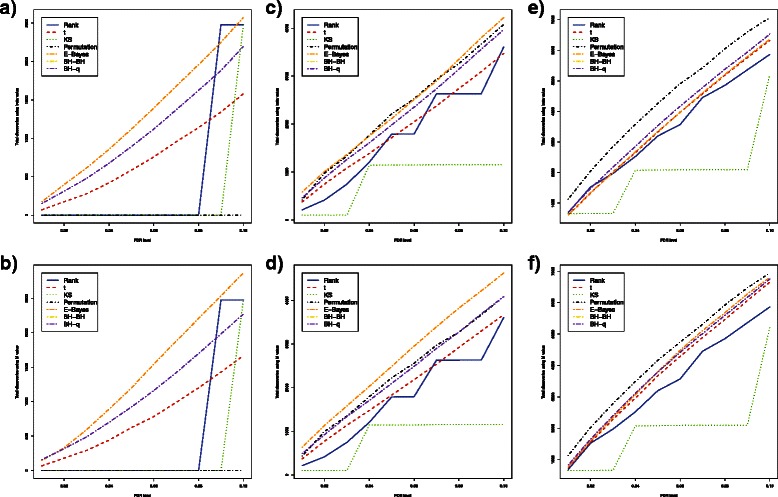
Total discoveries of the six DNA methylation differential analysis methods using both *β* and *M* values for methylation 27k data. Blue solid: rank test; Red dashed: *t*-test; Green dotted: KS test; Black dotdash: permutation test; Orange twodash: Empirical Bayes; Yellow twodash: Bump Hunting BH adjustment (BH-BH); Purple twodash: Bump Hunting *q*-value adjustment (BH-*q*)

When we randomly took 3 samples from both the cancer and control groups, both the empirical Bayes method and the bump hunting method showed higher apparent test power than the four other methods (Fig. 5). No discoveries were made either with the Wilcoxon rank sum test, the Kolmogorov-Smirnov test, or the permutation test, below a FDR level of 0.08. The *t*-test had lower apparent test power than the empirical Bayes method and the bump hunting method. However, differences between the empirical Bayes method and the bump hunting method were not significant. When we randomly selected 6 samples from both groups, total discoveries were similar for all methods, except for the Kolmogorov-Smirnov test which had still a relatively lower apparent test power than all other methods. No significant differences were observed between results using either *β*-values or *M*-values. When increasing sample size further to 12 in each group, we observed that all methods had more convergent apparent test power than when using a sample size of 6 in each group. Similarly, the Kolmogorov-Smirnov test showed increased power as sample size increased to 12 in each group.

#### Rheumatoid arthritis

Rheumatoid arthritis is a complex disease whose etiology involves the interaction of genetic, environmental, and life-style factors [27]. Epigenome-wide associations study have implicated DNA methylation as an intermediary of genetic risk in rheumatoid arthritis using Illumina HumanMethylation450 arrays on 354 rheumatoid arthritis cases and 337 controls [24]. The Methylation data was downloaded from the NCBI GEO website with accession number GSE42861. To demonstrate further the differences in apparent test power across the six methods at different FDR levels for popular HumanMethylation450 arrays, we randomly selected samples of size 3, 6, or 12 from both the rheumatoid arthritis case group and control group with the same FDR level set in the Ovarian Cancer example. The Storey’s *q*-value procedure and the Benjamini-Hochberg procedure from the bump hunting method had the same raw *p*-values.

**Fig. 6:**
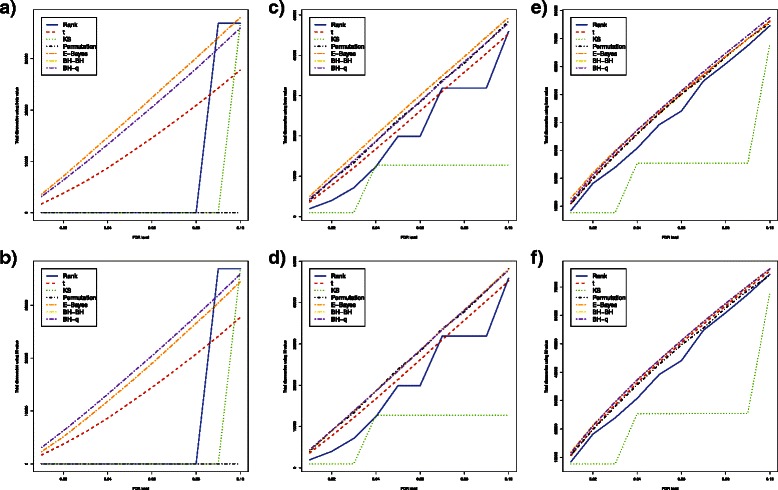
Total discoveries of the six DNA methylation differential analysis methods using both *β* and *M* values for methylation 450k data. Blue solid: rank test; Red dashed: *t*-test; Green dotted: KS test; Black dotdash: permutation test; Orange twodash: Empirical Bayes; Yellow twodash: Bump Hunting BH adjustment (BH-BH); Purple twodash: Bump Hunting *q*-value adjustment (BH-*q*)

The apparent test power showed similar results to those observed in the ovarian cancer example (Fig. 6). When sample size was 3 in each group, the empirical Bayes method and the bump hunting method had higher apparent test power than all other methods compared. The empirical Bayes method had a slightly higher apparent test power than the bump hunting method when *β* values were used, while the bump hunting method had a slighter higher apparent test power than the empirical Bayes method when *M*-values were used. All other methods showed similar results to those observed in the ovarian cancer example. When sample size was further increased to 6 or 12 in each group, the apparent test power of all methods compared were similar.

Overall, the results of the apparent test power comparisons of the six DNA methylation differential analysis methods using real data were consistent with our simulation results.

## Discussion and conclusions

In simulation studies, we compared six DNA methylation data analysis methods in terms of FDR control, power, and stability in both independent and correlated cases. These methods’ apparent test power based on raw *p*-values were also compared using two real data examples. For independent cases, no significant differences were detected between *β* values and *M* values in terms of FDR control, power, and stability, except that FDR was controlled at a lower level for the empirical Bayes method when *β* values were used for analysis. The similarity of the simulation results using either the *β* values or *M* values was probably due to the linear relationship between *β* and *M* values when *β* values were in the [0.2, 0.8] range and *M* values were in the [-2, 2] range as pointed out by Du [9]. The differences between *β* and *M* values in the empirical Bayes approach are likely a result of model mis-specification in the case of *β* values, potentially leading to an overestimation of standard deviations and thus deflation of significance. For correlated cases, the FDR control, power, and stability of the methods compared showed differences when using *β* values versus *M* values, which might have resulted from the correlations across CpG loci. The higher power and slightly lower stability observed in the *t*-test, the empirical Bayes method, and the bump hunting method when using *β* values rather than *M* values for small sample size data deserves further exploration.

In high-throughput data analysis, small or medium sample sizes are very common due to scant resources and funding constraints. Low statistical power challenges the reliability of studies, especially in small biomedical studies with low sample size [28]. Choosing appropriate approaches for DNA methylation data analysis could help investigators maximize the likelihood of true discoveries from small sample size studies with limited resources and funding. For small sample size data, both the empirical Bayes method and the bump hunting method showed good FDR control and much higher power than all other methods in independent cases. The empirical Bayes approach shrinks the estimated sample variance of the ordinary *t*-statistic towards a pooled estimate, resulting in higher power and more stable inference in small sample size studies [19]. The bump hunting method borrowed the strength of neighbor CpG loci, which improved the power of the DNA methylation analysis for small sample size [20]. When the methylation levels were correlated across CpG loci, only the bump hunting method showed decent control of FDR and much higher power than all other methods compared. The inflated FDR from the *t*-test and the empirical Bayes method was likely caused by the violation of the *t* distribution assumption when sample size is small, and by the violation of the independence assumption of methylation levels across CpG loci. The well-controlled FDR and high power from the bump hunting method might be due to its strength in taking the probe location information into account to model the correlation structure of error variances [20]. When sample size is very small (*n*=3), the zero power of the permutation test is due to the limited number of the possible combinations of permutation [29]. For the Wilcoxon rank sum test and the Kolmogorov-Smirnov test, the zero power when sample size is 3 may also be due to the discrete distribution of their test statistics estimated under the null hypothesis. For medium or large sample size, all methods had almost equivalent power, except for the permutation test with a very low proportion of differentially methylated loci, which deserves further exploration to elucidate causality. It is expected to see that the Storey’s *q*-value adjustment from the bump hunting method has higher power than the Benjamini-Hochberg’s *p*-value adjustment from the bump hunting method, as the Storey’s *q*-value adjustment has a larger cutoff value than the Benjamini-Hochberg *p*-value adjustment [22]. Meanwhile, the larger cutoff value from the Storey’s *q*-value adjustment resulting from the inclusion of estimated *π*_0_ also brought more variability in the analysis results, as indicated by the greater standard deviation of total discoveries.

In high-throughput data analysis, it is important to examine the power and stability of multiple testing procedures to learn the likelihood of true discoveries from empirical studies [30, 31]. In general, the use of either *β* values or *M* values is appropriate; however, it is advisable to take into account the differences observed between the *β* values and *M* values whenever applying the methods to DNA methylation differential analysis. When DNA methylation levels are independent across CpG loci, we recommend the bump hunting method and the empirical Bayes method in studies constrained by small sample sizes. When DNA methylation levels are correlated across CpG loci, we do recommend the bump hunting method in studies constrained by small sample sizes. In studies with medium to large sample size, all methods are suitable. With DNA differential methylation data analysis, researchers need to exercise caution with regard to the low stability of the bump hunting method whenever the proportions of differentially methylated loci are large, and with regard to the inflated FDR of the empirical Bayes method whenever DNA methylation levels are correlated across CpG loci in studies constrained by small sample sizes.

## References

[1] Baylin SB (2005). DNA methylation and gene silencing in cancer. Nat Clin Prac Oncol.

[2] Vavouri T, Lehner B (2012). Human genes with CpG island promoters have a distinct transcription-associated chromatin organization. Genome Biol.

[3] Das PM, Singal R (2004). DNA methylation and cancer. J Clin Oncol.

[4] Kaminskas E, Farrell A, Abraham S (2005). Approval summary: azacitidine for treatment of myelodysplastic dyndrome subtypes. Clinical Cancer Res.

[5] Sharma S, Kelly T, Jones P (2010). Epigenetics in cancer. Carcinogenesis.

[6] Moshe S. DNA methylation signatures for breast cancer classification and prognosis. Genome Med. 2012; 4(3):26. doi:10.1186/gm325. http://www.genomemedicine.com/content/4/3/26. 10.1186/gm325 PMC3446276 22494847

[7] Bibikova M, Barnes B, Tsan C, Ho V, Klotzle B, Le JM, Delano D, Zhang L, Schroth GP, Gunderson KL, Fan J, Shen R (2011). High density DNA methylation array with single CpG site resolution. Genomics.

[8] Wilhelm-Benartzi CS, Koestler DC, Karagas MR, Flanagan JM, Christensen BC, Kelsey KT, Marsit CJ, Houseman EA, Brown R (2013). Review of processing and analysis methods for DNA methylation array data. Br J Cancer.

[9] Du P, Zhang X, Huang C, Jafari N, Kibbe WA, Hou L, Lin SM (2010). Comparison of Beta-value and M-value methods for quantifying methylation levels by microarray analysis. BMC Bioinformatics.

[10] Price EM, Cotton AM, Lam LL, Farré P, Emberly E, Brown CJ, Robinson WP, Kobor MS. Additional annotation enhances potential for biologically-relevant analysis of the illumina infinium humanmethylation450 beadchip array. Epigenetics & Chromatin. 2013; 6(1):4. doi:10.1186/1756-8935-6-4. http://www.epigeneticsandchromatin.com/content/6/1/4. 10.1186/1756-8935-6-4 PMC3740789 23452981

[11] Benjamini Y, Hochberg Y (1995). Controlling the false discovery rate: A practical and powerful approach to multiple testing. J R Stat Soc Series B (Methodological).

[12] Storey JD (2002). A direct approach to false discovery rates. J R Stat Soc Series B (Methodological).

[13] Rubin D, Dudoit S, van der Laan MJ. A method to increase the power of multiple testing procedures through sample splitting. Stat Appl Genet Mol Biol. 2006; 5(1):1544–6115. http://www.degruyter.com/view/j/sagmb.2006.5.1/sagmb.2006.5.1.1148/sagmb.2006.5.1.1148.xml;jsessionid=2327FB240E9E1522A22AABE4359FABC9. 10.2202/1544-6115.1148 17049030

[14] Wilcoxon F (1945). Individual comparisons by ranking methods. Biometrics Bull.

[15] Rice JA (2006). Mathematical Statistics and Data Analysis, Third Edition.

[16] Kolmogorov A (1933). Sulla determinazione empirica di una legge di distribuzione. G Ist Ital Attuari.

[17] Smirnov N (1948). Table for estimating the goodness of fit of empirical distributions. Ann Math Stat.

[18] Westfall PH, Stanley Young S (1993). Resampling-Based Multiple Testing: Examples and Methods for p-Value Adjustment.

[19] Smyth GK (2004). Linear models and empirical bayes for asessingdifferential expression in microarray experiments. Stat Appl Genet Mol Biol.

[20] Jaffe AE, Murakami P, Lee H, Leek JT, Fallin MD, Feinberg AP, Irizarry RA (2012). Bump hunting to identify differentially methylated regions in epigenetic epidemiology studies. Int J Epidemiol.

[21] Aryee MJ, Jaffe AE, Corrada-Bravo H, Ladd-Acosta C, Feinberg AP, Hansen KD, Irizarry RA (2014). Minfi: a flexible and comprehensive bioconductor package for the analysis of infinium DNA methylation microarrays. Bioinformatics.

[22] Storey JD (2003). The positive false discovery rate: a Bayesian interpretation and the q-value. Ann Stat.

[23] Teschendorff AE, Menon U, Gentry-Maharaj A, Ramus S (2010). Age-dependent DNA methylation of genes that are suppressed in stem cells is a hallmark of cancer. Genome Res.

[24] Liu Y, Aryee M, Padyukov L, Fallin M (2013). Epigenome-wide association data implicate DNA methylation as an intermediary of genetic risk in rheumatoid arthritis. Nat Biotechnol.

[25] Houseman EA, Molitor J, Marsit CJ (2014). Reference-free cell mixture adjustments in analysis of DNA methylation data. Bioinformatics.

[26] Jemal A, Siegel R, Xu J, Ward E (2010). Cancer statistics. CA Cancer J Clin.

[27] Klareskog L, Catrina A, Paget S (2009). Rheumatoid arthritis. Lancet.

[28] Button KS, Ioannidis JP, Mokrysz C, Nosek BA, Flint J, Robinson ES, Munafò MR (2013). Power failure: why small sample size undermines the reliability of neuroscience. Nat Rev Neurosci.

[29] Li D, Dye TD (2013). Power and stability properties of resampling-based multiple testing procedures with applications to gene oncology studies. Comput Math Methods Med.

[30] Qiu X, Xiao Y, Gordon A, Yakovlev A (2006). Assessing stability of gene selection in microarray data analysis. BMC Bioinformatics.

[31] Gordon A, Glazko G, Qiu X, Yakovlev A (2007). Control of the mean number of false discoveries, bonferroni and stability of multiple testing. Ann Appl Stat.

